# High-accuracy quantitative principle of a new compact digital PCR equipment: Lab On An Array

**DOI:** 10.5808/gi.21035

**Published:** 2021-09-30

**Authors:** Haeun Lee, Cherl-Joon Lee, Dong Hee Kim, Chun-Sung Cho, Wonseok Shin, Kyudong Han

**Affiliations:** 1 Department of Bioconvergence Engineering, Dankook University, Yongin 16890, Korea; 2 Department of Anesthesiology and Pain Management, Dankook University Hospital, Cheonan 31116, Korea; 3 Department of Neurosurgery, Dankook University College of Medicine, Cheonan 31116, Korea; 4 NGS Clinical Laboratory, Dankook University Hospital, Cheonan 31116, Korea; 5 Center for Bio Medical Engineering Core Facility, Dankook University, Cheonan 31116, Korea; 6 Department of Microbiology, College of Science and Technology, Dankook University, Cheonan 31116, Korea

**Keywords:** digital PCR, LOAA dPCR, Micro Electro Mechanical System, point of care testing, Poisson distribution

## Abstract

Digital PCR (dPCR) is the third-generation PCR that enables real-time absolute quantification without reference materials. Recently, global diagnosis companies have developed new dPCR equipment. In line with the development, the Lab On An Array (LOAA) dPCR analyzer (Optolane) was launched last year. The LOAA dPCR is a semiconductor chip-based separation PCR type equipment. The LOAA dPCR includes Micro Electro Mechanical System that can be injected by partitioning the target gene into 56 to 20,000 wells. The amount of target gene per wells is digitized to 0 or 1 as the number of well gradually increases to 20,000 wells because its principle follows Poisson distribution, which allows the LOAA dPCR to perform precise absolute quantification. LOAA determined region of interest first prior to dPCR operation. To exclude invalid wells for the quantification, the LOAA dPCR has applied various filtering methods using brightness, slope, baseline, and noise filters. As the coronavirus disease 2019 has now spread around the world, needs for diagnostic equipment of point of care testing (POCT) are increasing. The LOAA dPCR is expected to be suitable for POCT diagnosis due to its compact size and high accuracy. Here, we describe the quantitative principle of the LOAA dPCR and suggest that it can be applied to various fields.

## Introduction

Digital PCR (dPCR) assay, a third-generation PCR method, is the latest PCR method capable of real-time absolute quantification of target genes without reference materials [1]. Commercial companies in the molecular diagnostic field have developed various dPCR systems because they have advantages over the existing quantitative PCR (qPCR) in several ways [2]. Following the evolving trend of this technique, a new dPCR equipment (Lab On An Array [LOAA] digital real-time PCR analyzer; LOAA dPCR, Optolane, Seongnam, Korea) was launched in South Korea last year. The LOAA dPCR is a separation type dPCR using semiconductor chip-based Micro Electro Mechanical System (MEMS) technology [3]. This LOAA dPCR equipment has several technical advantages. The amplification of the target gene and the fluorescence analysis of each well can be sequentially performed in only one device. For this reason, the false-negative probability is low because the effect of carry-over contamination is negligible.

**Fig. 1.**
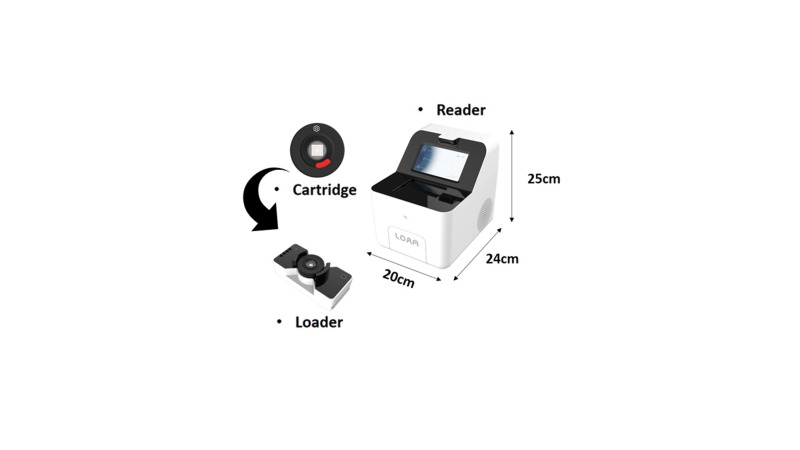
The components and size of the Lab On An Array (LOAA) digital PCR (dPCR). The LOAA dPCR has a compact size with length 24 cm, width 20 cm, and height 25 cm.

As the coronavirus disease of 2019 (COVID-19) has now spread worldwide, diagnostic equipment capable of point of care testing (POCT) in various diagnostic spaces is required [4,5]. Currently, COVID-19 diagnosis is carried out through qPCR equipment, which is less mobile because it consists of a thermal control device and a light source scanning device. Compared to other PCR systems, the semiconductor cartridge used in the LOAA dPCR is combined with the thermal control unit within the fluorescent sensor part, making it possible to dramatically reduce the equipment size (Fig. 1). These suggest that LOAA dPCR has potential in POCT with advantages of compact and highly accurate absolute quantification.

In 2019, the LOAA dPCR was approved as a class II medical device in South Korea. In addition, it was approved as an emergency use product that Viral Load 20K kit (Optolane) diagnose COVID-19. Here, we describe the principle of the LOAA dPCR, which can be quantified with high accuracy, and the POCT potential of this equipment with various advantages.

## Highly Accurate Quantification Principle of the LOAA dPCR

**Fig. 2.**
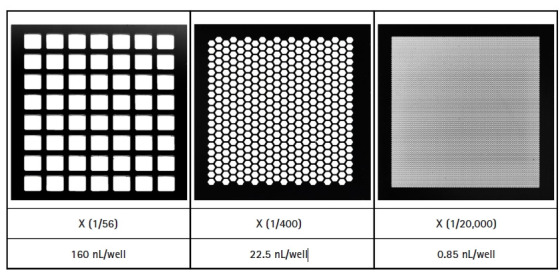
Micro Electro Mechanical System structures with 56 wells, 400 wells, and 20,000 wells. As the number of the well increases, the size of well gets partitioned into smaller volumes.

**Fig. 3.**
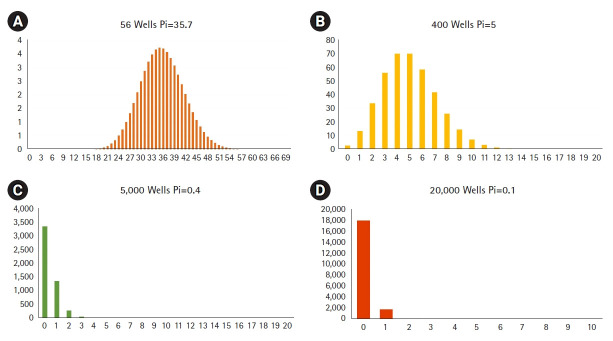
Poisson probability distribution when injecting 2,000 copy genes in 56 wells (A), 400 wells (B), 5,000 wells (C), and 20,000 wells (D). As the number of well increases from 56 wells to 20,000 wells, 99.5% of wells are digitized into 0 or 1.

The LOAA dPCR follows the semiconductor plate method using MEMS technology with excellent performance such as small size, light weight, rapid response, and precise measurement [3]. The MEMS structure can be injected by dividing the same amount of target molecules into as few as 56 wells and as much as about 20,000 wells. As the number of wells increases, the size of well is gradually partitioned into smaller volumes (Fig. 2). To verify that LOAA dPCR follows Poisson distribution statistics, we confirmed the four types of Poisson distribution when injecting 2,000 copy genes in 56 wells, 400 wells, 5,000 wells, and 20,000 wells [1]. As a result, we could identify that when the number of wells increases, 99.5% of wells are digitized to 0 or 1 (Fig. 3). In other words, the LOAA dPCR allows absolute quantification because it follows the Poisson distribution containing either 0 or 1 molecules in the wells divided into the partitions.

Micro-statistics, based on Monte Carlo Simulation [6], which randomly places target molecules with temporal and spatial equivalences, are represented by Bose-Einstein Statistics. Then it becomes equal to a random photon in a sensor array without physical effect. The statistical standard deviation of the target gene based on the mean value, called photon noise, can be represented as Eq. 1.

(1) $$\sigma_{SHOT}(P_{I}{)}^{2}=P_{I}\frac{e_{\mathrm{kT}}^{E}}{e_{\mathrm{kT}}^{E}-1}$$

*P_I_* means the average number of photons. E is the energy value of the photon. *K* stands for Boltzmann’s constant (1.38 × 10^-23^ J/K), and *T* refers to the absolute temperature. When *E*≫*kT*, it is expressed as Eq. 2 which is the shot noise characteristic.

(2) $$\sigma_{SHOT}(P_{I})=\sqrt{P_{I}}$$

The shot noise distribution above can be described by the classical Poisson distribution shown below, where *p_i_* represents the probability that there are *i* target genes per well.

(3) $$p_{i}=\frac{P_{I}^{i}}{i!}e^{-P_{I}}$$

The graph would appear in Gaussian distribution form if *p_i_* increases as the number of wells decreases (Fig. 3). This means as the *p_i_* increases, the standard deviation increases.

(4) $$p_{0}=\frac{the\;number\;of\;negative\;PCR\;well}{the\;total\;number\;of\;available\;PCR\;well}$$

On the other hand, in the dPCR, *p*_0_ can be calculated using the Poisson probability distribution represented in Eq. 5.

(5) $$p_{0}=e^{-PI}$$

*P_I_* represents the average number of target molecules injected per well and can be specifically expressed as Eq. 6, according to the experiment definition.

(6) $$P_{I}=\frac{the\;number\;of\;target\;genes\;in\;well(x)}{the\;total\;number\;of\;available\;PCR\;well(AW)}$$

Applying Eqs. 4, 5, and 6, *p*_0_, the probability that no target molecule is in the well, can be expressed as Eq. 7.

(7) $$\frac{the\;number\;of\;negative\;PCR\;well}{the\;total\;number\;of\;available\;PCR\;well}=e^{-\frac{x}{AW}}$$

If we calculate *x*, the first number of target genes in a sample, it can be expressed as Eq. 8.

(8) $$x=-(the\;number\;of\;available\;pcr\;well)\times log_{e}(\frac{the\;number\;of\;PCR\;well}{the\;total\;number\;of\;available\;PCR\;well})$$

Finally, we can confirm the accuracy of the LOAA dPCR by analyzing the Poisson distribution of Eqs. 2 and 6.

(9) $$\sigma_{SHOT}(P_{I})=\sqrt{P_{I}}=\sqrt{\frac{the\;number\;of\;target\;genes\;in\;well}{the\;total\;number\;of\;available\;PCR\;well}}$$

(10) the number of target genes in well ≪ thelnumberloflavailablelPCRlwell

When the condition Eq. 10 is met, the standard deviation becomes small. In other words, the accuracy of the LOAA PCR increases when the amount of target gene decreases.

## Region of Interest Selection

**Fig. 4.**
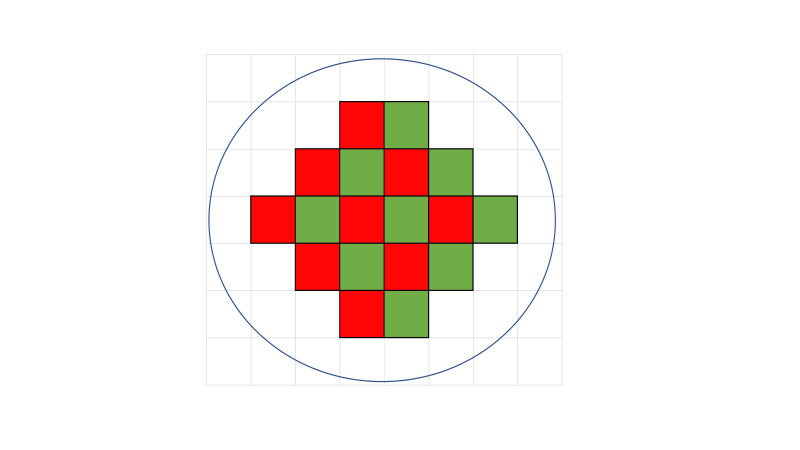
Well region of interest with two channels. When there are two channels (FAM/FRET-Cy-5), data is generated using an average of six samples.

The first and important step in sample detection prior to PCR is to locate the region of interest within the cartridge divided into 20,000 wells. A way to differentiate between valid wells and invalid wells is to examine Otsu Filter to investigate pixel differences [7]. When there is one channel (1 channel: FAM), well data is formed based on the average value of 12 pixels. On the other hand, if there are two channels (2 channels: FAM/FRET-Cy5), the data is generated using the average of each six samples (Fig. 4).

## Invalid Well Filtering Methods

**Fig. 5.**
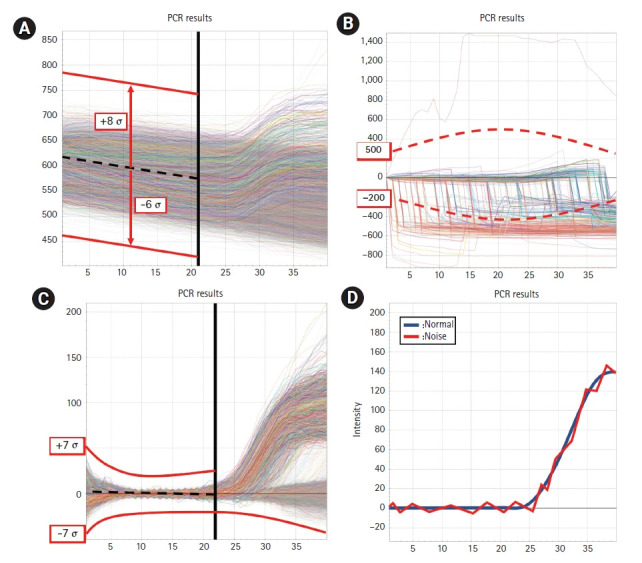
Filtering methods. (A) In the brightness filter threshold, data exceeding two multiples (‒6 σ to 8 σ) within 22 cycles is considered invalid. (B) For the slope filter threshold, data exceeding 10 multiples (‒200 to 500) is judged invalid. (C) The baseline filter threshold is based on the negative cycle without the target gene. If the data is less than -7 σ or exceeds 7 σ, it is considered invalid. (D) Normal and noise graph. Depending on the algorithm, when the curve is not continuous, it is judged to be noise.

When plotting a histogram using acquired well data after the run ends, the data is generally distributed from 3 σ below to 4 σ above of the mean. For brightness filter, data exceeding two multiples (-6 σ to 8 σ) in 1 to 22 cycles is judged to be invalid (Fig. 5A).

In the entire PCR cycle, the slope of available well data is generally between 20 and 50. Considering the noise data that occurs at the beginning of the run, the data is excluded if the slope exceeds 10 multiples (‒200 to 500) (Fig. 5B). In addition, for the baseline filter, invalid wells can be determined by negative wells which have none of the target genes. Negative well cycle data below -7 σ and above 7 σ is treated invalid. Because when the threshold range is narrowed, the noise well and a number of normal wells are considered non-functional, resulting in well loss (Fig. 5C).

Detecting invalid well also involves filtering noise based on the algorithm written in Eq. 11. If the curve is not continuous, it is considered unavailable (Fig. 5D)

(11) $$AccNoise\;=\;\frac{\Sigma cycle((Noise-Normal{)}^{2})}{total\;cycle}$$

## Discussion

Since the COVID-19 pandemic has occurred, many countries have come to require equipment capable of POCT diagnosis. POCT equipment requires two conditions: size and sensitivity. Compared with existing commercialized qPCR and dPCR devices in the diagnostic market, the LOAA dPCR has a relatively compact size. In addition, the LOAA dPCR has twice the detection sensitivity of DNA and three times the sensitivity of RNA compared to Bio-rad equipment, called gold-standard equipment [8]. Therefore, there is a growing interest in compact and accurate diagnostic equipment such as LOAA dPCR.

Accuracy and sensitivity of the LOAA dPCR may contribute to the advancement of GMO tests, drug resistance research, and personalized cancer treatment [9]. LOAA dPCR is a prominent potential candidate for a comparative data method as a reference data in the clinical area by detecting infectious diseases caused by viruses and bacteria [10].

The LOAA dPCR has a compact size due to its structural specificity and has high accuracy based on the Poisson statistics. Based on these advantages, it is expected to be utilized in more various fields.
